# APOBEC3B Does Not Promote Tumor Progression in *Tp53* Hemizygous Mice

**DOI:** 10.1002/cnr2.70189

**Published:** 2025-04-11

**Authors:** Yoshihito Horisawa, Tadahiko Matsumoto, June Takeda, Yusuke Tashiro, Ryosuke Nomura, Suguru Takeuchi, Yugo Kawai, Yasuhiro Kazuma, Yoshinobu Konishi, Hiroyuki Yamazaki, Hiroyuki Matsui, Kotaro Shirakawa, Akifumi Takaori‐Kondo

**Affiliations:** ^1^ Department of Hematology Graduate School of Medicine, Kyoto University Kyoto Japan

**Keywords:** APOBEC3B, cancer, somatic mutation, Tp53, transgenic mouse

## Abstract

**Background:**

DNA cytosine deaminase APOBEC3B (A3B) is one of the endogenous sources of somatic mutations in many types of human cancers and is associated with tumor progression rather than tumorigenesis. However, it remains uncertain whether APOBEC3B‐induced mutations accelerate tumor progression or not. In this paper, we established a mouse model with A3B overexpression and investigated whether the introduction of A3B overexpression accelerates tumor development in *Tp53* hemizygous mice.

**Methods:**

We established A3B transgenic mouse by microinjection and selected the mouse which has only one A3B transgene by genomic qPCR and southern blotting using the probe against the transgene. A3B expression was validated by qPCR, immunoblotting, immunohistochemistry and *in vitro* CDA assays using lysates of this transgenic mouse liver, spleen and bone marrow. We interbreed this transgenic mouse model with CAG‐Cre and *Tp53* knockout mice and observed differences in tumor progression and survival between *Tp53* hemizygous mice and *Tp53* homozygous mice irrespective of A3B expression. Finally, comprehensive genomic mutation analysis was done using the developed tumors.

**Results:**

We established A3B transgenic mouse which has only one transgene. A3B expression and its CDA activity were confirmed in liver cells and tumor tissues of mice overexpressing A3B. *Tp53* hemizygous mice developed osteosarcomas, spindle and pleomorphic sarcomas, and squamous cell carcinomas, however we did not observe any difference in tumor development between the mice with or without A3B expression. The tumor with A3B expression has more high‐VAF mutations than the one without A3B, but these mutations are not APOBEC signature.

**Conclusion:**

We developed a Cre inducible A3B transgenic mouse model bearing single copy of *A3B* gene. Although the introduction of A3B overexpression did not accelerate tumor development in *Tp53* hemizygous mice, our mouse model with A3B overexpression is well‐validated and useful for further research.

**Abbreviations**
APOBEC: apolipoprotein B mRNA editing enzyme catalytic polypeptide‐like
A3B: APOBEC3B
CDA: cytidine deaminase assays

## 1 Introduction

The human Apolipoprotein B mRNA‐editing enzyme catalytic polypeptide‐like 3 (APOBEC3) family consists of seven proteins, APOBEC3A/B/C/D/F/G/H, and most of them have cytosine deaminase (CDA) activity, which converts cytosine to uracil in single‐stranded DNA. APOBEC3 proteins were first reported to suppress retroviral infection and retrotranspositions, but this suppression is not only dependent on their CDA activity [1, 2]. Recent whole exome or genome sequence studies identified that a substantial amount of the mutations that are specific to APOBEC3 proteins were accumulated in many types of cancers and have been shown to be one of the endogenous sources of somatic mutations in many types of human cancers [3]. Such specific patterns of mutations are called mutation signatures, and some of the mutation signatures are assumed to be due to APOBECs [4]. Since the APOBEC‐signature mutation load was higher in the patients with aggressive myeloma than in smoldering myeloma patients, APOBEC mutations are thought to be associated with tumor progression as well as tumorigenesis [5] or more contributed to tumor progression in esophageal cancer [6].

Among these APOBEC3 family proteins, we focused on APOBEC3B (A3B) because tumors with increased expression of A3B have more amounts of APOBEC‐signature mutations. This finding implies that, among APOBEC proteins, A3B could be the major source of these mutations. We previously reported that endogenous A3B generates DNA substitutions in the genomic DNA of multiple myeloma cell lines [7]. A3B is transcribed by canonical [8] and non‐canonical [9] NF‐kB pathways, and DNA damage response pathway activation leads to its expression in myeloma cell lines [7, 10]. Protein kinase A suppresses the CDA activity of A3B via its phosphorylation [11]. p53 suppresses A3B expression by recruiting the DREAM complex at the A3B promoter region, and deletion of p53 in tumor cells enhances A3B expression and promotes its CDA activity [12, 13]. The catalytic activity of A3B is also regulated by its interaction with ILF2. ILF2 binds to A3B and enhances its CDA activity [14].

Many reports showed that A3B induces lots of genomic mutations in cells, but the mechanisms of how A3B induces genomic mutations are still unclear. Since A3B can bind and catalyze single‐stranded DNA (ss‐DNA), not double‐stranded DNA (ds‐DNA), genomic DNA needs to be ssDNA to become A3B's substrate. It is reported that A3B catalyzes ssDNA derived from DNA double‐strand break repair and R‐loop during transcription [15]. It is also unknown whether some mechanisms are required for A3B recruitment in ssDNA, like another APOBEC family protein, AID.

We experimentally proved that A3B catalyzes cytosine on genomic DNA and introduces C‐to‐T mutations [16]. However, we have not known if A3B induces genomic mutations in vivo, what pattern of mutations they are, or if such accumulated mutations result in tumor formation. To address this question, we established a mouse model with conditional A3B expression using the Cre/LoxP system. This mouse model was well validated by A3B expression and its CDA activity using mouse tissue. We interbred this transgenic mouse model with CAG‐Cre and *Tp53* knockout mice and observed differences in tumor progression and survival between *Tp53* hemizygous mice and *Tp53* homozygous mice irrespective of A3B expression. Although the tumor expresses A3B, we could not see any effect of A3B on tumor progression or survival. In this model, we failed to show that overexpression of A3B accelerates the tumor development of the *Tp53* hemizygous mouse; we successfully established a well‐validated mouse model of conditional A3B expression, and this mouse model is available for future studies.

## 2 Materials and Methods

### 2.1 Animal Experiments

We obtained male C57BL/6J mice (controls) from CLEA Japan Inc. (Tokyo, Japan). The p53 KO mouse and CAG‐Cre mouse were previously described [17]. We established an A3B transgenic mouse by microinjecting a linearized LoxP‐EGFP‐LoxP‐A3B‐3xFLAG vector into fertilized eggs. Genotyping PCR of the mice was performed as described below. We lysed mouse tails with HotShot reagent (25 mM NaOH, 0.2 mM EDTA) for 30 min at 95°C, followed by adding one‐twenty‐fifth amount of 1 M Tris–HCl (pH 8). Then the genotype was determined by genotyping PCR at EGFP loci (EGFP‐Fw: CGTGCTGGTTATTGTGCTGTC, EGFP‐Rv: TAGGTCAGGGTGGTCACGAG). We also determined mouse genotype by EGFP positivity by flow cytometry of mouse peripheral blood. Genotyping PCR was performed on mice generated by crossing A3B mice with CAG‐Cre mice and by crossing A3B‐expressing mice with p53 knockout mice, using the following primers: A3B‐F; A3B‐R; Cre‐Fw GTTTCACTGGTTATGCGGGCGG; Cre‐Rv TTCCAGGGCGCGCGAGTTGATAG; p53‐KO‐Fw GTTATGCATCCATACAGTACA; p53‐WTKO‐Rv ACACCCAACACCATACCATGT; p53‐Rv TCTGGATTCATCGACTGTGG. Genotypes, the date of birth, and death for all mice used for the survival analysis are provided in Table S2.

### 2.2 Plasmids

We introduced intron5 of A3B into the coding sequence of A3B by PCR reaction. we first amplified the 5′ portion and 3′ portion of A3B using primers: 5′ Fw: NNNNgctagcCACCATGAATCCACAGATCAGAAATCCG, 5′ Rv: GATGCAGGTGGCTGGGTCGGTCACctcgttgcatagaaagcccatgtg, 3′ Fw: CACACTCTGTTTCCTTTTCTAGgctaagaatcttctctgtggcttttacg, 3′ Rv: NNNNctcgagTCAGTTTCCCTGATTCTGGAGAATG. A3B Intron 5 was amplified by PCR from genomic DNA using primers: 5′ intron5: cacatgggctttctatgcaacgagGTGACCGACCCAGCCACCTGCATC, 3′ intron5: cgtaaaagccacagagaagattcttagcCTAGAAAAGGAAACAGAGTGTG. Then, we purified these PCR products and did PCR using the mixture of these three PCR products as a template with 5′ Fw and 3′ Rv primers. Then, the A3B intron5 PCR product was subcloned into the Nhe1‐Xho1 site of the LoxP‐EGFP‐LoxP‐MCS‐pA vector.

### 2.3 Southern Blotting

We lysed mouse tail with tail lysis buffer (50 mM Tris–HCl (pH 8.0), 100 mM NaCl, 20 mM EDTA, 1% SDS, 0.4 mg/mL Proteinase K) at 55°C overnight and isolated genomic DNA by the phenol‐chloroform method. Then, 5 mg of the purified genomic DNA was treated with BamHI at 37°C overnight and was run in 1% agarose gel. We denatured the gel in denature buffer (0.5 M NaOH, 1.5 M NaCl), neutralized it, and then the genomic DNA was capillary‐transferred to a nitrocellulose membrane overnight. Then, the membrane was cross‐linked by UV and hybridized with DIG‐labeled EGFP probe. In brief, the PCR product for the probe was amplified from the transgene vector (Probe Fw: CCCGCGCCGAGGTGAAGTTC, Probe Rv: GTTTTACTTGCTTTAAAAAACCTCCC) and DIG‐nylated using DIG‐High Prime DNA Labeling and Detection Starter Kit II (Roche, Cat# 11585614910) following the manufacturer's protocol. The membrane was visualized by X‐ray film.

### 2.4 qPCR

Mouse tissues were harvested, crushed into small clumps, and filtered through a cell strainer. ACK lysis buffer was added and incubated for 2 min to lyse red blood cells before storage in TRIzol RNA Isolation Reagents. RNA was extracted from mouse tissues using the miRNeasy Mini kit (Qiagen). Complementary DNA was synthesized using ReverTra Ace qPCR RT Master Mix with gDNA Remover (TOYOBO). Real‐time PCR was performed using TB Green Premix Ex Taq II (Takara). Target gene expression levels were normalized by endogenous expression levels of HPRT1, and then the relative expression levels of A3B were obtained by calculating the relative expression of A3B to the average expression level of A3B in A3B^−^ mice. A3B and HRPT1 transcript was amplified with the following primers: A3B‐F GACCCTTTGGTCCTTCGAC; A3B‐R GCACAGCCCCAGGAGAAG; HPRT‐F TGGCCATCTGCCTAGTAAAGG; HPRT‐R GGCTCATAGTGCAAATCAAAAGTC. qPCR was performed using two mice, and statistical analysis was done by t‐test. *p*‐values < 0.05 were considered statistically significant.

### 2.5 Western Blotting

Mice normal or tumor tissues were homogenized in a lysis buffer (2% SDS, 4 M Urea, 1 mM EDTA, 150 mM NaCl, 50 mM Tris; pH 8.0) by sonication, denatured by heating at 95°C for 5 min. For co‐immunoprecipitation assays, the lysates were immunoprecipitated using the ANTI‐FLAG M2 Affinity Gel (A2220 Sigma‐Aldrich) at 4°C for 4 h with RNase A, DNase‐free, followed by immunoblotting. Equal amounts of protein were used for SDS‐polyacrylamide gel electrophoresis. Proteins were transferred from the gel to the Immobilon PVDF membrane (Merck Millipore). Anti‐FLAG antibody (F3165, Sigma‐Aldrich) and Anti‐βactin (A5441, Sigma‐Aldrich) were used for immunoblotting. Immunoblots were detected with ECL Prime Western Blotting Detection Reagent and Amersham ImageQuant LAS 800 (Cytiva).

### 2.6 Gel‐Based CDA Assays

Mouse liver or tumor lysate was made by sonicating these tissues in GST‐lysis buffer (25 mM HEPES‐NaOH pH 7.4, 150 mM NaCl, 0.5% Triton‐X, 1 mM MgCl_2_ , 1 mM ZnCl_2_ , 10% Glycerol, 1 mM EDTA) and protein concentration was measured with the kit. We incubated each protein lysate with 1 pM of single‐strand DNA oligonucleotide (ATTATTATTATTCAAATGGATTTATTTATTTATTTATTTATTT) with 5’‐FAM, 0.005 units of UDG, and 3.75 μL of reaction buffer in a 10 μL reaction volume for 2 h at 37°C. Then, the oligo products were incubated in 100 mM NaOH for 30 min at 37°C, denatured at 95°C for 3 min, and separated in a 20% TBE/urea‐acrylamide gel, which was then visualized with the Gel Doc EZ Gel Documentation System (Bio‐Rad).

### 2.7 Immunohistochemistry

We snap‐froze mouse liver or tumor tissue in OCT (Sakura Finetek) on dry ice. We sectioned the frozen tissues with a cryostat and fixed them with 4% PFA for 15 min at room temperature. Then, the tissue was blocked in 5% BSA, incubated with ANTI‐FLAG antibody produced in rabbit (F7425 Millipore) for 1 h in 5% BSA, followed by incubation with the N‐Histofine MOUSESTAIN KIT (NICHIREI BIOSCIENCES) for 1 h.

### 2.8 Whole Exome Sequencing

We analyzed paired tumor and germline DNA from *Tp53* hemizygous tumors with or without A3B overexpression using whole exome sequencing (WES). Briefly, tumor and germline DNA was extracted from the tumor and from the tail, respectively, using the QuickGene DNA tissue kit S (DT‐S KURABO) according to the manufacturer's instructions. Samples were subjected to massively parallel sequencing with 150 bp paired‐end reads using the Illumina HiSeq according to the manufacturer's instructions. Sequencing reads were aligned to the NCBI mouse reference genome mm10 (m38) by Burrows−Wheeler Aligner, with default parameters. PCR duplicates were eliminated using Picard tools version 3.v4 (GATK). Mutation calling was performed using Mutect2 [18].

### 2.9 Statistical Analysis

We performed survival analysis using the Kaplan–Meier method. Overall Survival (OS) was calculated using all the mice for which their genotype can be determined Event Free Survival (EFS) was defined by the duration from the birth to the date of tumor development or death from any other reason. Cumulative incidence of tumor development was reckoned by the duration from birth to tumor development. The log‐rank test was applied to compare Kaplan–Meier curves. Statistical analyses were performed using Microsoft Excel 2000 and R 4.1.1 (The R Foundation for Statistical Computing).

## 3 Results

### 3.1 Generation of Human A3B Transgenic Mice in *Tp53* Hemizygous Background

**FIGURE 1:**
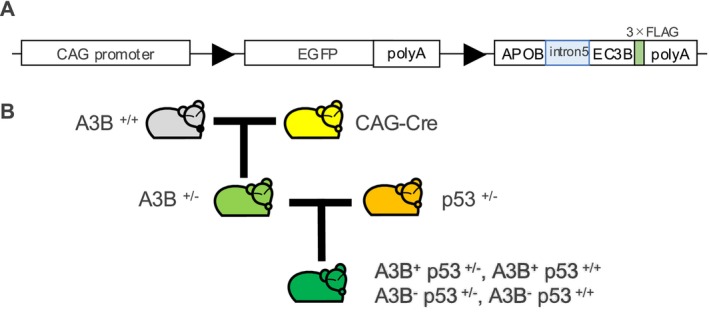
Generation of human A3B expression transgenic mice in p53 KO. (A) Schema of constructs of lentiviral vector. A3B transgene includes Intron 5. (B) Breeding schema. Mating homozygous A3B^+^ male mice with female CAG‐Cre mice generates A3B^+^ Cre^−^ and A3B^+^ Cre^+^ mice with Cre‐mediated induction of A3B expression. A3B^+^ mice are selected and crossed with *Tp53* hemizygous mice to generate *Tp53* hemizygous mice that express A3B systemically.

To create conditional transgenic mice expressing A3B, we included an EGFP‐stop cassette franked by LoxP sequence between a CAG promoter and A3B‐3xFLAG sequence (Figure 1A), so we can identify EGFP positive cells containing the transgene and control A3B expression under Cre expression. We finally obtained four GFP positive mice out of 36 mice born after microinjection and chose the mouse with a single copy of the transgene to avoid unwanted Cre‐induced translocation between two different transgenes. To determine the copy number of the transgene in each mouse, we employed southern blot and successfully established the mouse #13 with a single‐copy transgene (Figure S1). Since A3B reportedly relates to tumor progression more than tumor initiation, we planned to observe the effect of A3B on top of the p53 hemizygous KO model, which develops sarcomas and other epithelial cancers within a year of age [19]. We chose this model because the overexpression of A3B results in cell growth arrest induced by DNA damage in vitro, and such DNA damage‐induced cell growth arrest mostly depends on p53 [20, 21]. To generate A3B‐Cre mice that overexpress A3B in the whole mouse body, we first crossed homozygous A3B transgenic male mice with female heterozygous CAG‐Cre mice. Next, we crossed A3B‐expressing mice with p53 heterozygous knockout mice to generate four genotypes: A3B^+^ p53^+/**−**^, A3B^+^ p53^+/+^, A3B^**−**^ p53^+/**−**^, A3B^**−**^ p53^+/+^ (Figure 1B).

### 3.2 Validation of A3B Expression and Deaminase Activity in Normal Tissue

**FIGURE 2:**
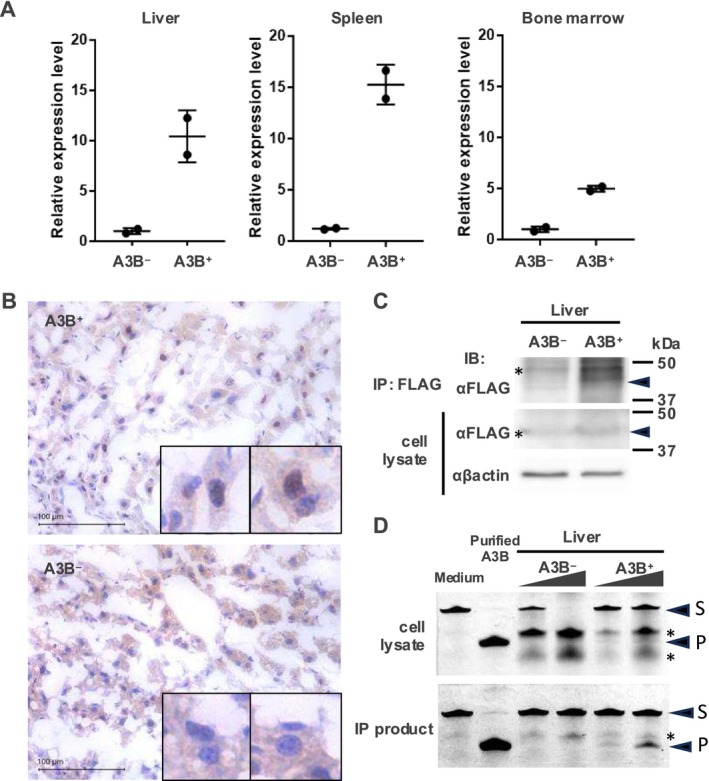
Validation of A3B expression in normal tissue. (A) qPCR analysis of A3B mRNA expression in Cre positive (A3B^+^) and Cre negative (A3B^−^) mice using the organs indicated. A3B mRNA level is higher in A3B^+^ mice than in A3B^−^ mice. (B) Immunostaining of frozen liver tissue using anti‐FLAG antibody, top panel: A3B^+^, bottom panel: A3B^−^. Black bars in the left lower corner indicate 100 μm. (C) Immunoblots of liver from A3B^+^ p53^+/−^ mice and A3B^−^ p53^+/−^ mice. Arrowheads indicate A3B detected by anti‐FLAG antibody. Asterisks indicate non‐specific bands. Immunoprecipitation using anti‐FLAG antibody made the A3B band much clearer than that in the cell lysate. (D) TBE urea PAGE analysis of A3B CDA assay from A3B^+^ p53^+/−^ mice and A3B^−^ p53^+/−^ mouse. Arrowhead labeled “S” indicates substrates; arrowhead labeled “P” indicates products. Asterisks indicate non‐specific degradation of the substrate. Liver lysate from A3B^−^ mice did not show any CDA activity, whereas that from A3B^+^ showed substantial CDA activity.

We sought to confirm whether A3B is indeed expressed in mice crossed with A3B transgenic mice and CAG‐Cre mice. We first checked A3B mRNA expression by RT‐qPCR in the liver, spleen, and bone marrow of homozygous A3B transgenic mice and A3B‐Cre mice. A significant upregulation of A3B mRNA in A3B‐Cre mice was observed (Figure 2A, Table S1). Next, we checked A3B protein expression using histological and biochemical methods. Immunostaining using an anti‐FLAG antibody detected A3B in the nuclei of liver cells from a frozen section of the liver of a mouse expressing A3B (Figure 2B). However, it was unclear if A3B is expressed by immunoblotting of liver samples from tumor‐forming A3B^+^ p53^+/−^ and A3B^−^ p53^+/−^ mice because of a non‐specific band that is close to the A3B band (Figure 2C, bottom panel). Then, we performed immunoprecipitation of A3B with an anti‐FLAG antibody to enrich A3B protein and to remove the non‐specific band. Immunoprecipitation made the A3B band (Figure 2C, upper panel) much clearer than the one from the cell lysate, and there is a clear difference between A3B‐ and A3B+ mice.

Since still in vitro CDA assays are the gold standard to confirm the A3B protein expression, we performed a gel‐based CDA assay. Overall, 43 base length ss‐DNA substrates (substrates) labeled with FAM at the 5′ end were A3B CDA activity substrates. Subsequent cytosine to uracil transfer, uracil excision, and cleavage of the newly formed abasic site result in the formation of a 12‐base‐long 5′ FAM‐labeled oligo DNA (product) [22]. We used purified A3B C‐terminal domain protein as a positive control and buffer as a negative control. First, we tested liver cell lysates and detected CDA activity, but most of the cleaved oligo DNA was non‐specific cleavage (Figure 2D, upper panel, asterisks), suggesting that cell lysates contain endogenous nucleases which could generate these non‐specific bands. To eliminate cellular contamination, we did this assay using an immunoprecipitated A3B sample. The lysates from each mouse were immunoprecipitated with anti‐FLAG antibody, and we could see clear product bands in the A3B expressing sample (Figure 2D, bottom). These findings suggest that the A3B gene under the CAG promoter is expressed and has deaminase activity, which could possibly cause somatic mutations by cytosine deamination in mouse tissues.

### 3.3 A3B Expression Does Not Affect Onset of Tumors or Mouse Survival Under Hemizygous *Tp53* Background

**FIGURE 3:**
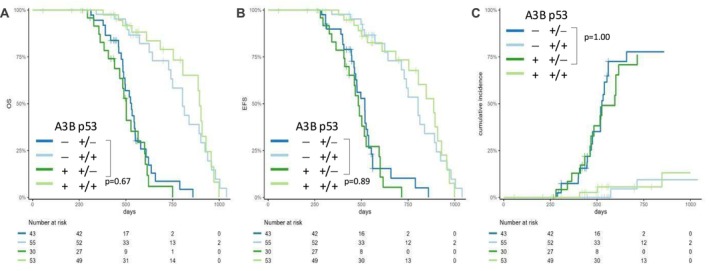
A3B expression does not cause tumor promotion under p53 KO. Kaplan–Meier analysis of (A) overall survival (OS), (B) event‐free survival (EFS), and (C) cumulative incidence (CI) of tumor development for A3B^+^ p53^+/−^, A3B^+^ p53^+/+^, A3B^−^ p53^+/−^, and A3B^−^ p53^+/+^ mice. Median follow‐up time was 433 d (133–999 d). Statistical significance was determined by a log‐rank test. A3B expression did not affect survival on the top of p53^+/−^ or p53^+/+^ background.

To investigate how overexpressing A3B affects tumor formation and progression, we crossed A3B‐Cre mice with p53^+/−^ mice to generate four genotypes of A3B^+^ p53^+/−^, A3B^+^ p53^+/+^, A3B^−^ p53^+/−^, and A3B^−^ p53^+/+^, and observed their tumor formation and their survival. Comparing A3B^+^ p53^+/+^ mice with A3B^−^ p53^+/+^ mice, most mice showed no tumor formation and no significant differences in overall survival (OS), event‐free survival (EFS), or cumulative incidence of tumor development (Figure 3A–C, Table S2). This suggests that A3B expression did not cause tumorigenesis under p53 WT conditions.

On the other hand, most of the hemizygous *Tp53* mice developed tumors, but there were no significant differences in OS, EFS, or cumulative incidence of tumor development between A3B^+^ p53^+/−^ and A3B^−^ p53^+/−^ mice (Figure 3A–C), indicating that A3B overexpression did not affect the onset of tumors and did not cause tumor promotion under a hemizygous *Tp53* background.

### 3.4 Validation of A3B Expression and Deaminase Activity in the Tumor

**FIGURE 4:**
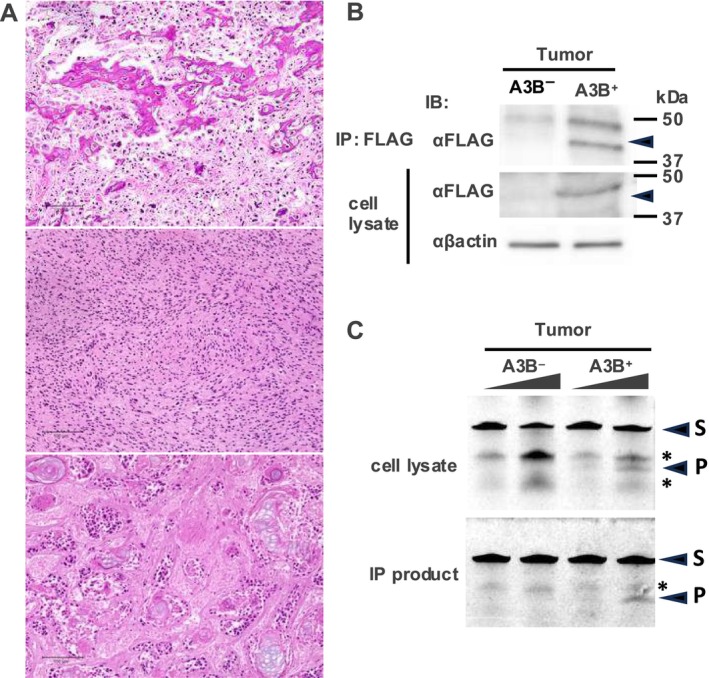
Validation of A3B expression in tumor. (A) Photomicrographs of H&E‐stained tumor tissue from A3B‐expressing p53 KO mice. Pathological findings of osteosarcoma (upper panel), spindle and pleomorphic sarcoma (middle panel), and squamous cell carcinoma (lower panel) are shown. (B) Immunoblots of tumor cells from A3B^+^ p53 KO^+/−^ mice and A3B^−^ p53^+/−^ mice. (C) TBE urea PAGE analysis of A3B CDA assay from A3B^+^ p53 KO^+/−^ mice and A3B^−^ p53^+/−^ mice. S indicates substrates, P indicates products. Asterisks indicate non‐specific degradation of the substrate.

Histopathological examination defined that tumors developed in the hemizygous *Tp53* background included osteosarcomas, spindle and pleomorphic sarcomas, and squamous cell carcinomas (Figure 4A, top to bottom panels). To investigate the A3B expression and its deaminase activity in tumor tissue, we performed immunoprecipitation experiments using an anti‐FLAG antibody to test Western blotting and CDA assays. By using immunoprecipitation lysates from osteosarcoma samples in A3B^+^ mice, A3B expression was detected in immunoblotting (Figure 4B), and deaminase activity was detected in the CDA assay (Figure 4C). These data indicated that tumor tissue from A3B^+^ p53^+/−^ mice had no tumor‐promoting effect, even though A3B protein was expressed and showed deaminase activity in the tumor tissue as well as in the normal organ tissue.

### 3.5 Whole Exome Sequencing of *Tp53* Hemizygous Tumor With or Without A3B Overexpression In Vivo

**FIGURE 5:**
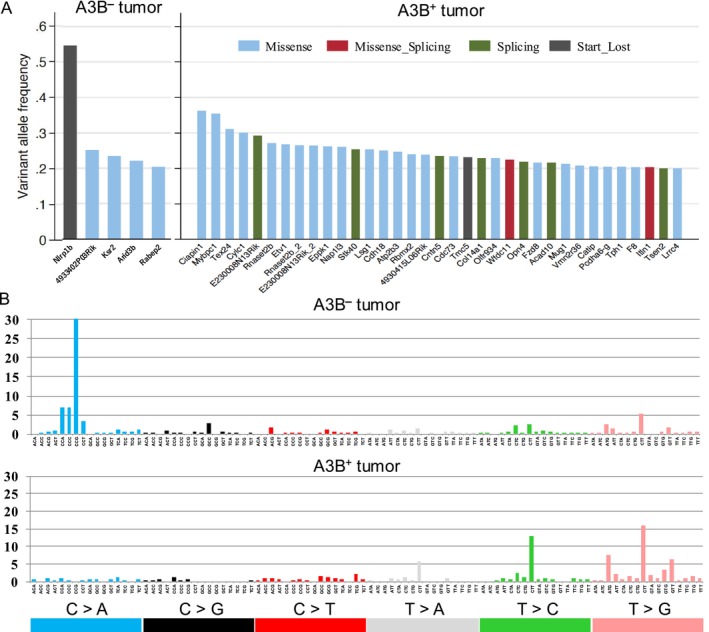
Whole exome sequencing of *Tp53* hemizygous tumor with or without A3B overexpression in vivo. (A) Genes mutated with high VAF, more than 0.2 in A3B^**−**^ tumor (left panel) and A3B^+^ tumor (right panel). (B) Mutation signature of A3B^**−**^ tumor (upper panel) and A3B^+^ tumor (lower panel). Note that APOBEC signature mutations include C > T or C > G mutations in mainly TCA trinucleotide patterns.

To evaluate if A3B overexpression affected the genomic mutation profile, we used Illumina HiSeq to perform whole exome sequencing of sarcoma samples of Tp53 hemizygous background with or without A3B overexpression. We used tail DNA as controls and Mutect2 to call somatic mutations. Sequencing depths were more than x100 for sarcoma samples and more than ×60 for control tail samples. We detected a total of 571 and 332 mutations for A3B^**−**^ tumors and A3B^+^ tumors, respectively. Interestingly, mutations with high VAF (variant allele frequency) (more than or equal to 0.2) were 6 in A3B^**−**^ tumors and 35 in A3B^+^ tumors (Figure 5A, left and right panels). We then analyzed mutational signature using all detected SNVs and found that A3B overexpression did not induce mutations of the APOBEC signature (Figure 5B, upper and lower panel). These data suggest that A3B overexpression somehow contributed to the accumulation of DNA mutations.

## 4 Discussion

In this study, we generated conditional A3B transgenic mice under the Cre/LoxP system and developed A3B overexpressing mice with a *Tp53* hemizygous background. We confirmed that A3B expression and its deaminase activity in the normal tissue and the developed tumor, but A3B overexpression has no effects on tumor development, tumor size, or tumor histology on top of *Tp53* heterozygous knockout in our mouse model.

Recently, one report showed that overexpression of A3B promoted tumor formation in a mouse model [23], and another report showed that EGFR tyrosine kinase inhibitor resistance is induced by NF‐κB induced A3B expression in a mouse model [24]. In the former paper, the mouse model with better A3B expression developed tumors, whereas one with a lower expression level did not, so it is suggested that the expression level of A3B should be important for the development of tumors. Since our model has a low A3B expression level as indicated by immunoblot and in vitro CDA assays, this must be the reason why our model did not promote cancer development. In the latter paper, A3B overexpression inhibited tumor initiation in the EGFR mutant mouse model. Although both reports showed that A3B overexpression induced APOBEC pattern mutations in developed tumors.

Since APOBEC targets cytosine in ss‐DNA, APOBEC is reported to induce mutations in single‐stranded regions generated during DNA repair and DNA replication [20, 25, 26]. We hypothesized that decreased expression of p53 might enhance the development of APOBEC mutations by inhibiting cell cycle arrest after DNA damage, but our results showed that overexpressed A3B deaminase activity did not affect tumor development and formation. These results suggest several possibilities. First, DNA mutations induced by A3B may be repaired in DNA repair pathways. In fact, the high VAF mutations (VAF > 0.2) accumulated in the A3B expressing tumor. Second, overexpression of A3B deaminase activity may not accumulate the DNA mutations required for tumor progression in mouse cells, as A3B has been shown to be preferentially expressed in the G2/M phase of the cell cycle [27, 28, 29]. Decreased p53 expression may suppress cell cycle arrest during G2, thereby reducing A3B expression [30, 31, 32]. Third, the expression levels of A3B in our mouse tissue were not abundant enough to accumulate the DNA mutations and develop or promote tumors. Durfee et al. reported that only high A3B expression animals promoted tumor development by inducing APOBEC‐pattern mutations, but low A3B expression animals did not [23]. APOBEC1 overexpressing mice develop liver carcinomas, but these animals were reported to contain 3 to 17 transgenes [33]. Finally, it is possible that somatic mutations caused by A3B may simultaneously enhance tumor immunity as well as tumor progression. In some tumors, high A3B expression correlates with activation of tumor immunity, increased neoantigen load, and enhanced immunotherapy responsiveness [34, 35, 36].

In this study, we did not observe the effect of A3B on tumor progression nor development in sarcomas developed in the *Tp53* hemizygous background, but it is still possible that A3B contributes to tumor development in other cell types of tumors. Although most of our mouse models develop sarcomas, the A3B mutational signature is most frequently found in breast cancer, lung cancer, urinary cancer, head and neck cancer, and multiple myeloma, and such differences in the type of tumor could be the reason why we were unable to see the effect of A3B in tumor development. To overcome this problem, it might be worth testing the induction of A3B expression on top of other appropriate types of tumor models, like the XBP‐1 transgenic mouse [37], one of the representative myeloma mouse models. Alternatively, intercrossing with more specific DNA damage response‐deficient mice, such as the UNG KO mouse [38], might be useful to see the accumulation of A3B‐induced mutations and tumor development.

Taken together, we confirmed A3B expression at the transcript level and protein level, and its CDA activity in our A3B mouse model. Since mice have only one murine APOBEC3 protein and its subcellular localization is different from human A3B, we cannot analyze the biological or pathological functions of murine APOBEC3 as a counterpart of human A3B. Therefore, our mouse model could be very useful to investigate the in vivo effect of A3B on suppression of viral infection, like MMTV and MLV, or inhibition of retrotransposition, such as LINE‐1.

In conclusion, we developed a transgenic mouse model with conditional expression of A3B using the Cre‐LoxP system. These mice successfully expressed A3B after Cre‐induced recombination. Although we could not prove that overexpression of A3B had no effects on tumor progression, survival, and pathology on top of *Tp53* hemizygous KO, we established a well‐defined conditional A3B transgenic mouse model. This in vivo model could be very useful for further research to study cancer development, clonal evolution, and tumor immunity by crossing with other mouse models such as DNA repair deficient mice and could also be useful to test if A3B suppresses retrotransposition and viral infection.

## Author Contributions

Y. H., K. S., T. M., and A. T.‐K. conceived the study; Y. H. carried out experiments with help from Y. T., R. N., S. T., Y. K., Y. K., Y. K., H. M., H. Y., and T. M.; Y. H. and J. T. analyzed WES; Y. H., T. M., J. T., K. S., and A. T.‐K. wrote the paper. All the authors reviewed and approved the manuscript.

## Ethics Statement

Animal studies: All animal experiments were approved by the animal research committee of Kyoto University (approval number MedKyo 21 107).

## Conflicts of Interest

A.T.‐K. declares that this study received funding from ONO PHARMACEUTICAL CO. LTD. The funder was not involved in the study design, collection, analysis, interpretation of data, the writing of this article, or the decision to submit it for publication. Other authors declare no conflicts of interest.

## Supporting information


**Table S1.** qPCR raw data for Figure 2A.
**Table S2.** Survival data for Figure 3.

**Figure S1.** Southern blotting image of mouse genotyping.
**Figure S2C, D, S4B, C.** Full gel images for corresponding figures.

## Data Availability

The data that support the findings of this study are available from the corresponding author upon reasonable request.
