# Supplementary material for: APOBEC3B Does Not Promote Tumor Progression in Tp53 Hemizygous Mice
Source: Cancer Rep (Hoboken). 2025 Apr 11;8(4):e70189. doi: 10.1002/cnr2.70189 (PMC11986841; doi:10.1002/cnr2.70189)

Supplementary FIGURE 1

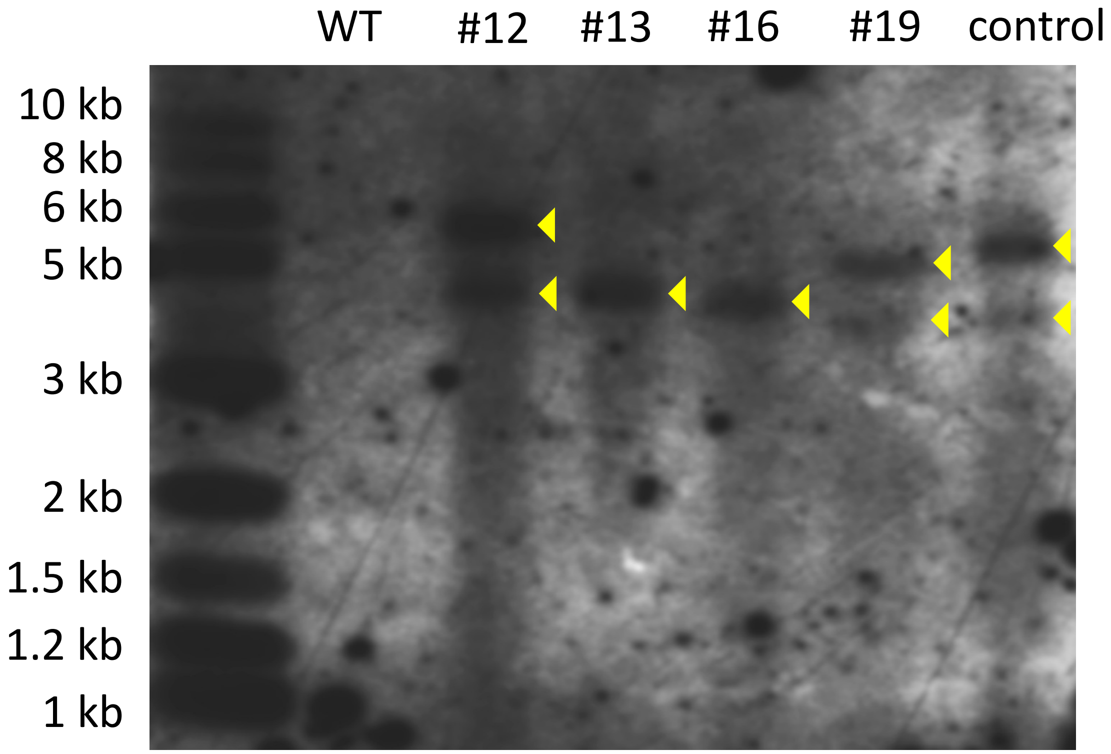

Supplementary FIGURE 2C

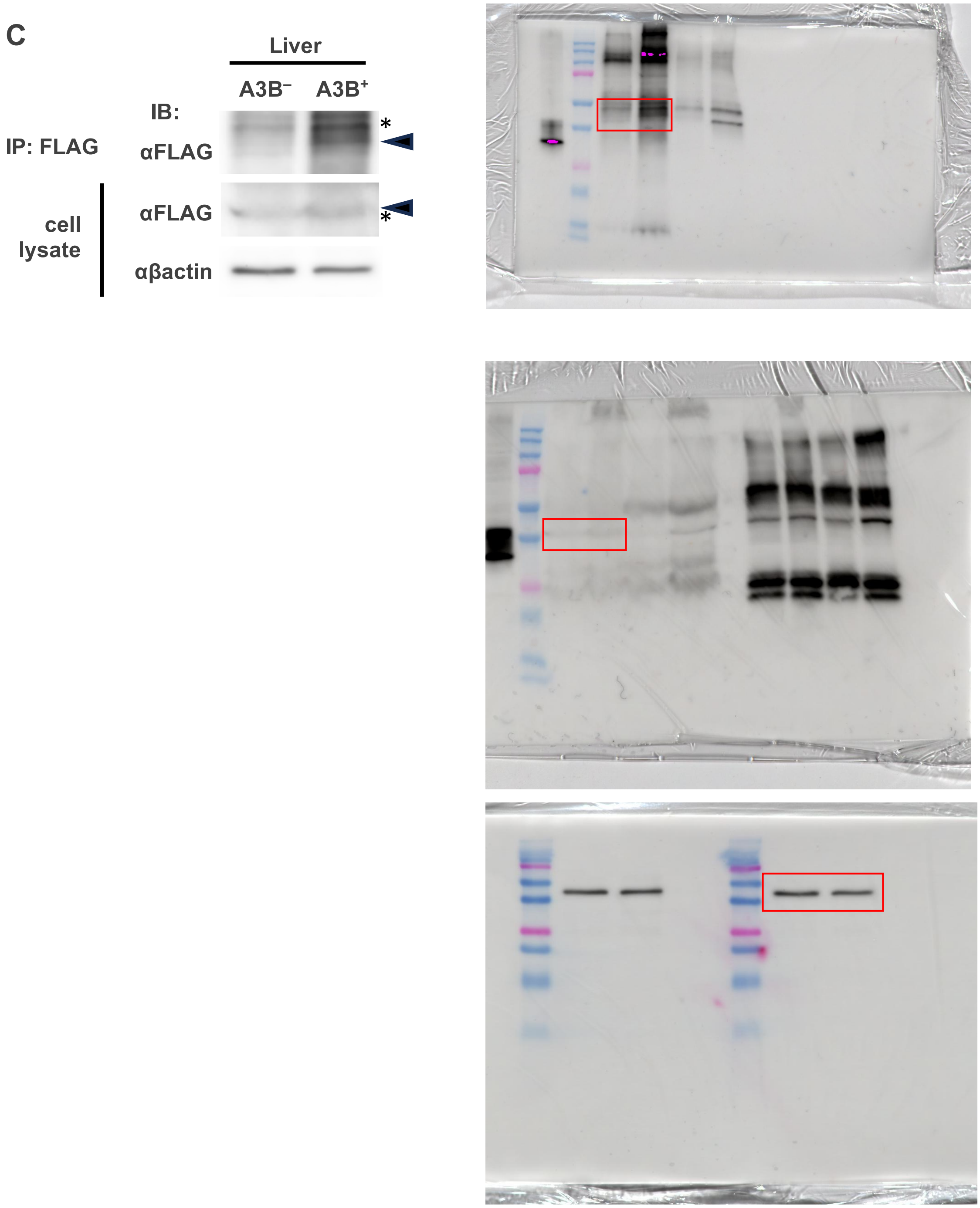

Supplementary FIGURE 2D

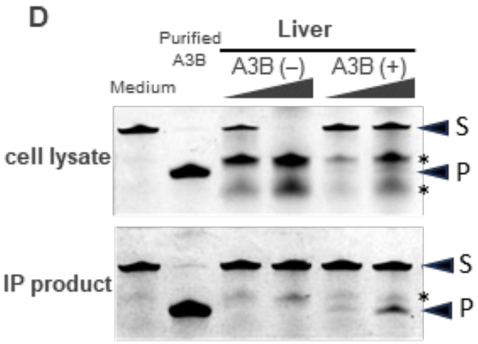

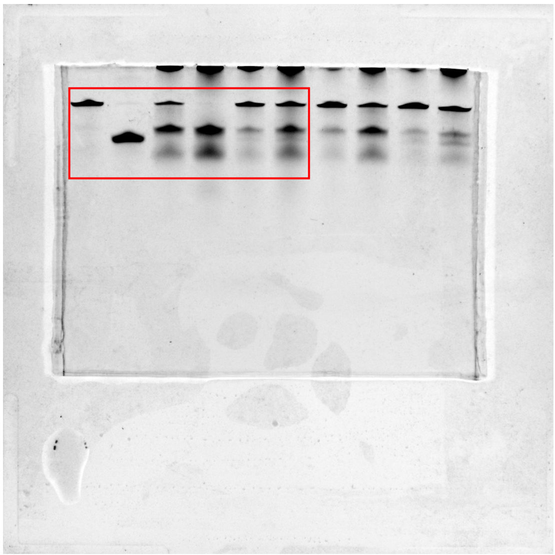

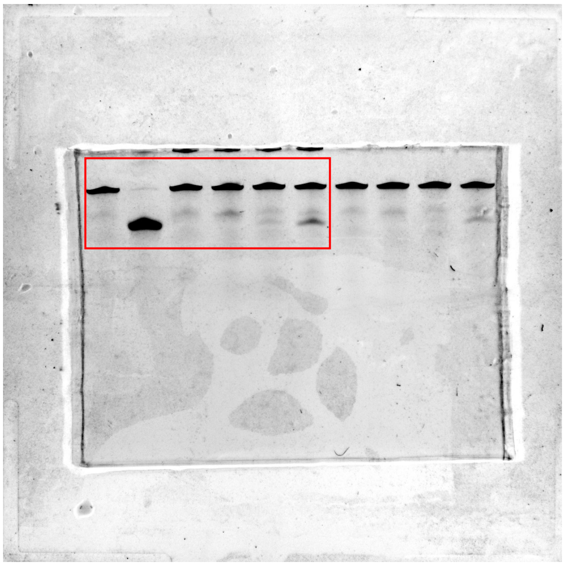

Supplementary FIGURE 4B

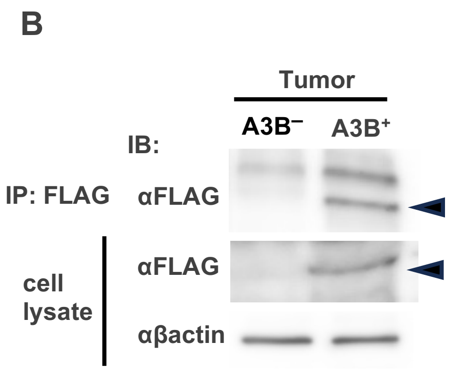

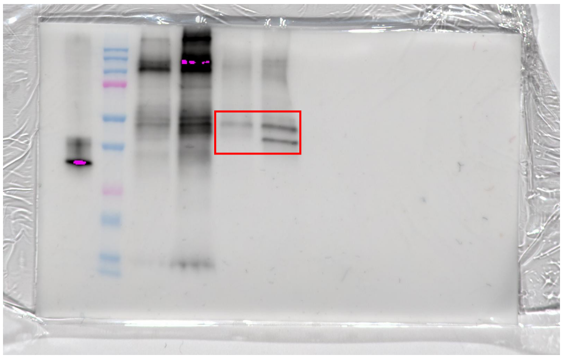

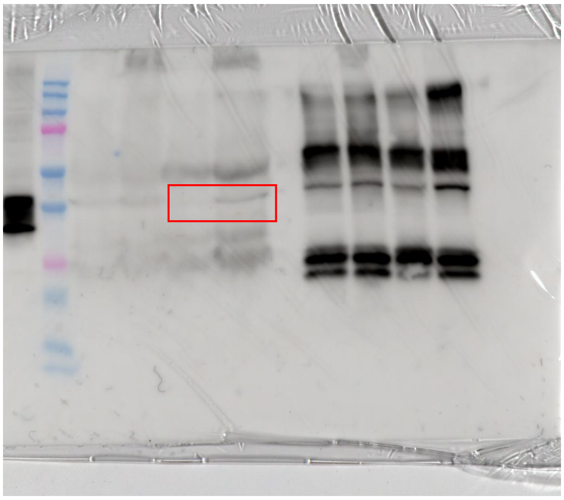

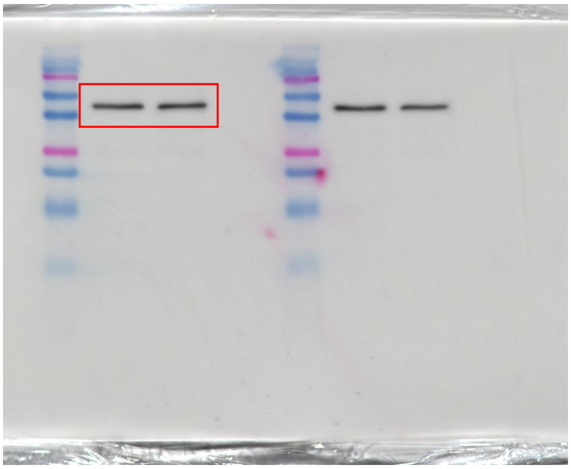

FIGURE 3C
Supplementary FIGURE 4C

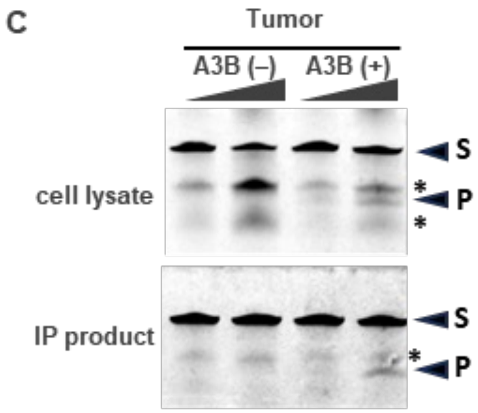

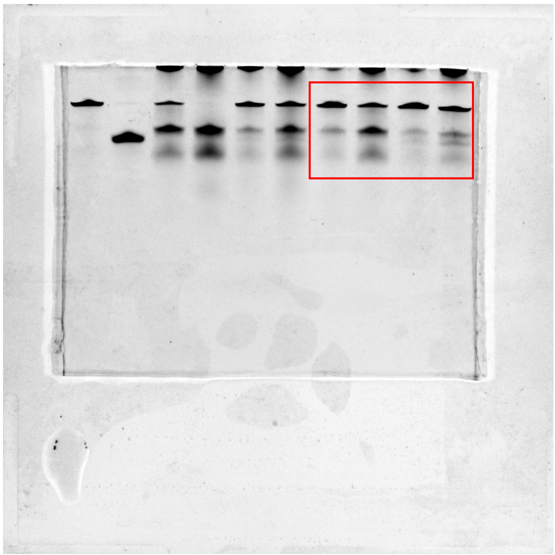

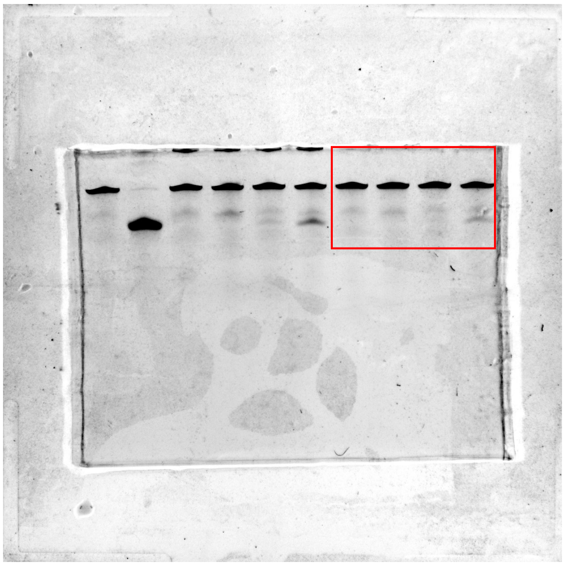

Supplement: Supplementary file 2 — Figure S1. Southern blotting image of mouse genotyping. Figure S2C, D, S4B, C. Full gel images for corresponding figures. [file CNR2-8-e70189-s001.pdf]
